# Rapid Detection of Extended-Spectrum β-Lactamases (ESBL) and AmpC β-Lactamases in *Enterobacterales*: Development of a Screening Panel Using the MALDI-TOF MS-Based Direct-on-Target Microdroplet Growth Assay

**DOI:** 10.3389/fmicb.2019.00013

**Published:** 2019-01-24

**Authors:** Carlos L. Correa-Martínez, Evgeny A. Idelevich, Katrin Sparbier, Markus Kostrzewa, Karsten Becker

**Affiliations:** ^1^Institute of Medical Microbiology, University Hospital Münster, Münster, Germany; ^2^Bruker Daltonik GmbH, Bremen, Germany

**Keywords:** extended-spectrum beta-lactamase, AmpC beta-lactamase, MALDI-TOF mass spectrometry, rapid diagnostic assays, multiresistance, *Enterobacterales*, *Enterobacteriaceae*, minimum inhibitory concentration

## Abstract

**Introduction:** Antibiotic resistant bacteria are a growing concern worldwide. Extended-spectrum β-lactamases (ESBL) represent the most common resistance mechanism of Gram-negative bacteria against β-lactams, underlining the need for adequate diagnostic methods that provide reliable information in the shortest time possible. AmpC, a less prevalent but increasingly relevant class of β-lactamases, pose an additional challenge as their detection is complex. Here, we present an ESBL and AmpC screening panel employing the MALDI-TOF MS-based direct-on-target microdroplet growth assay (DOT-MGA).

**Materials and Methods:** Four reference strains recommended by the European Committee on Antimicrobial Susceptibility Testing (EUCAST) were used to develop the panel, which was further validated on 50 clinical *Enterobacterales* isolates resistant to third generation cephalosporins. The panel relies on the synergistic effect between ESBL and/or AmpC β-lactamase inhibitors and cephalosporins, which indicates β-lactamase production. Microdroplets containing the tested microorganism, cephalosporins in different concentrations and inhibitors were pipetted onto an MBT Biotarget and incubated for 3 or 4 h at 35 ± 1°C. Afterward, the liquid medium was removed and the material adhered to the spots was analyzed by MALDI-TOF MS. Synergy was detected by determining and comparing the minimum inhibitory concentrations of the tested cephalosporins with and without β-lactamase inhibitors. Data were interpreted following a diagnostic algorithm proposed by EUCAST in order to establish a final diagnosis. In comparison, PCR, broth microdilution (BMD) and combination disk tests (CDT) were performed.

**Results:** Compared to the PCR results, the following positive and negative percent agreement values (PPA/NPA) were obtained for each resistance mechanism: ESBL, 94.44/100%; AmpC, 94.44/93.75% and ESBL+AmpC, 100/100%. These results, obtained after 4 h of incubation, were comparable with those of BMD and showed a higher accuracy than CDT.

**Discussion:** We propose a novel phenotypic method for detection of ESBL and AmpC β-lactamases in *Enterobacterales* that provides reliable results in a short time, representing a promising alternative to the diagnostic techniques currently available. This easy-to-perform approach has potential for being implemented in routine laboratories, contributing to the further diversification of mass spectrometry technology into other fields such as antibiotic resistance testing.

## Introduction

A new class of β-lactamases able to hydrolyze expanded-spectrum β-lactam antibiotics was first described in 1985 in a *Klebsiella pneumoniae* strain (Kliebe et al., 1985). By the end of the decade, a broad range of bacteria producing these enzymes could be found in healthcare facilities worldwide. Less than 20 years after their first identification, these microorganisms already represented one of the most important groups of nosocomial pathogens (Gniadkowski, 2001). Today, extended-spectrum β-lactamases (ESBL) are the most common resistance mechanism of Gram-negative bacteria against β-lactam antibiotics (Al-Bayssari et al., 2015) and have become a concern for public health, with growing infection and colonization rates worldwide (Karanika et al., 2016; McDanel et al., 2017). ESBL-producing bacteria have also been described to play an important role beyond the boundaries of the hospital setting, as indicated by the occurrence of community-associated infections in patients without discernible healthcare-associated risk factors (Coque et al., 2008; Doi et al., 2012). Moreover, high colonization rates among hospitalized and non-hospitalized individuals have been detected in several regions (Schaumburg et al., 2013; Köck et al., 2016), which brings the hidden burden of this problem into the light.

AmpC β-lactamases, which confer resistance against a broad range of substrates, are less prevalent than ESBL but still a growing issue, having been identified in several outbreaks (Roh et al., 2008; Mansouri et al., 2014; Uzunovic et al., 2014; Kameyama et al., 2015). Multiple factors contribute to the severity of this problem, including the fact that these enzymes confer resistance to carbapenems when combined with decreased outer membrane permeability (Philippon et al., 2002; Woodford et al., 2007) and that they are not neutralized by ESBL inhibitors, which limits the possible phenotypic diagnostic and therapeutic approaches. AmpC is chromosomally encoded in several common Gram-negative bacteria such as *Enterobacter* spp., *Citrobacter freundii*, or *Serratia marcescens*. Additionally, plasmid-encoded *ampC* genes can be horizontally transferred to other *Enterobacterales* with no chromosomally encoded AmpC such as *Klebsiella, Proteus*, and *Salmonella*, which represents a highly effective and dynamic mode of dissemination (Bauernfeind et al., 1998; Ingram et al., 2011). This underlines the importance of developing simple and valid detection methods for AmpC production, which are currently scarce (Reuland et al., 2015).

For the detection of ESBLs and AmpC β-lactamases, phenotypic and genotypic methods are employed. The latter, which include polymerase chain reaction (PCR) and next generation sequencing (NGS), have gained relevance in clinical laboratories in the last decades (Decousser et al., 2017). They allow for a highly accurate characterization of resistance mechanisms, being of great advantage in cases where phenotypic results are unclear. However, these methods must be performed by trained personnel and require facilities fully equipped with all necessary elements. This often translates into high costs, thus limiting the availability of such methods in routine laboratories. Moreover, unknown or not annotated variants will be missed.

Phenotypic approaches to detect ESBL and/or AmpC are based on the detection of synergy between β-lactam agents and specific substances that inhibit each enzyme type. In the broth microdilution (BMD), synergy is indicated by a substantial decrease of the minimum inhibitory concentration (MIC) of the β-lactam (Wiegand et al., 2008). Following this principle, several methods that employ disks containing β-lactams and inhibitors have been validated and are widely used (M’Zali et al., 2000; Nourrisson et al., 2015). Since these assays require overnight incubation, the turnaround time amounts to 18 h (Decousser et al., 2017). This delay is also the main disadvantage of other culture-based approaches such as the double-disk synergy test, three-dimensional tests, gradient diffusion tests as well as automated systems (Drieux et al., 2008). Rapid testing with disk diffusion has been described, however, being recommended only for preliminary susceptibility reports. It requires further standardization and adapted clinical breakpoints for a correct interpretation (Froding et al., 2017). Colorimetric methods represent a substantially faster alternative, although some of them display a low positive predictive value in the presence of AmpC hyperproduction (Decousser et al., 2017).

Methods based on matrix-assisted laser desorption ionization time-of-flight mass spectrometry (MALDI-TOF MS) can also be applied to identify β-lactam resistance. One of these approaches relies on the detection of products resulting from the hydrolysis of β-lactam antibiotics by β-lactamases (Sparbier et al., 2012; Oviano et al., 2017; Lee et al., 2018). Nonetheless, this depends on several factors that are specific to the enzyme-substrate interaction (such as enzyme availability), which could interfere with the results, as well as alternate mechanisms that also lead to misdetection of antibiotic hydrolysis. Moreover, a positive result indicates the presence of resistance, but does not provide an exact MIC value for the tested antibiotic (Decousser et al., 2017). Another strategy consists in detecting isotopically labeled amino acids, which requires a specific culture medium that can be supplemented with these substances (Sparbier et al., 2013; Jung et al., 2014). The applicability of techniques that analyze the amount of biomass resulting from bacterial incubation with antibiotic agents in order to establish susceptibility patterns has also been described (Sparbier et al., 2016).

Considering the need for rapid methods that are easy to standardize, we developed and validated a screening panel for detection of ESBL and AmpC β-lactamases in *Enterobacterales* adapting the principle of the MALDI-TOF MS-based direct-on-target microdroplet growth assay (DOT-MGA) previously described (Idelevich et al., 2018a,b). While this method does not rely on the detection of hydrolyzed β-lactam, it resembles the MIC determination by BMD. The panel’s layout and the interpretation criteria follow the criteria of the European Committee on Antimicrobial Susceptibility Testing (EUCAST) (EUCAST, 2017). With this approach, we sought to establish a method able to overcome common obstacles in the detection of these resistance mechanisms, such as unclear results in isolates producing both types of β-lactamases as well as false negative-results due to inadequate AmpC identification. The assay was validated on clinical isolates of the order *Enterobacterales* including species of the *Enterobacteriaceae, Hafniaceae, Morganellaceae*, and *Yersiniaceae* families to further assess its practicability and accuracy.

## Materials and Methods

### Bacterial Strains and Cultures

*Enterobacterales* strains were consecutively isolated from clinical samples processed in the routine diagnostic laboratory at the Institute of Medical Microbiology, University Hospital Münster, Germany. Species identification was performed by MALDI-TOF MS. Isolates displaying phenotypic resistance against third generation cephalosporins in the susceptibility testing performed routinely with Vitek 2^®^ (bioMérieux, Marcy-l’Étoile, France) were consecutively collected. In total, 50 strains were tested, 25 belonging to each of the two groups defined by EUCAST depending on the mechanism of resistance most likely involved (EUCAST, 2017): group 1, ESBL (*Escherichia coli, Klebsiella pneumoniae, K. oxytoca, Raoultella ornithinolytica*); group 2, AmpC (*Hafnia alvei, C. freundii, C. koseri, S. marcescens, Enterobacter cloacae* complex, *E. aerogenes, Morganella morganii*). As a reference, three resistant control strains recommended by EUCAST for the detection of ESBL and AmpC production were tested (*K. pneumoniae* ATCC 700603, *E. coli* CCUG 62975, *E. coli* CCUG 58543), as well as one negative control strain (*E. coli* ATCC 25922) (EUCAST, 2017). Bacterial suspensions were prepared using colonies grown on blood agar. Density was adjusted to 0.5 McFarland employing a nephelometer (Densimat, bioMérieux, Marcy-l’Étoile, France). Subsequently, a dilution 1:100 was made with cation-adjusted Mueller-Hinton broth (CA-MHB).

### DOT-MGA-Based Screening Panel

**FIGURE 1:**
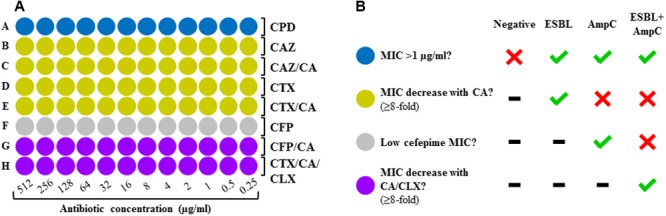
**(A)** Layout of the DOT-MGA screening panel. Blue zone, resistance screening; yellow zone, detection of ESBL; gray zone, detection of AmpC; purple zone, detection of ESBL + AmpC. CPD, cefpodoxime; CAZ, ceftazidime; CTX, cefotaxime; CFP, cefepime; CA, clavulanic acid; CLX, cloxacillin. **(B)** Interpretation of results according to EUCAST’s diagnostic criteria.

A screening panel was developed on a 96-spot format following the layout depicted on Figure 1A. The MIC of four cephalosporins was determined in absence and presence of an ESBL inhibitor (clavulanic acid, 4 μg/ml) and an AmpC inhibitor (cloxacillin, 512 μg/ml) in order to establish a result on the basis of the synergy observed. The layout was designed according to the detection algorithm suggested by EUCAST (2017). It comprises four zones: screening for resistance against third generation cephalosporins with cefpodoxim (blue zone), ESBL detection with cefotaxime and ceftazidime in presence and absence of ESBL inhibitor (yellow zone), AmpC detection with cefepime (gray zone), and detection of AmpC plus masked ESBL with cefepime plus ESBL inhibitor or cefotaxime plus ESBL inhibitor and AmpC inhibitor (purple zone).

#### Antibiotic Substances

Stock solutions were prepared following the guidelines of the Clinical and Laboratory Standards Institute (CLSI) (CLSI, 2018) by mixing the following antimicrobial agents in powder form with deionized distilled water: cefepime, cefpodoxime (TOKU-E, Bellingham, WA, United States), ceftazidime, potassium clavulanate (Sigma-Aldrich, Saint Louis, MO, United States), cefotaxime, cloxacillin (Tokyo Chemical Industry Co., LTD, Tokyo, Japan). Quality controls of the solutions were carried out in accordance with the specifications of the CLSI (2018) and EUCAST (2018).

#### MALDI-TOF MS

DOT-MGA was performed as previously described (Idelevich et al., 2018a). Briefly, microdroplets containing 3 μl of antibiotic solution and 3 μl of bacterial suspension (final inoculum approximately 5 × 10^5^ CFU/ml) were pipetted onto an MBT Biotarget 96 (Bruker Daltonik, Bremen, Germany). Sterility and growth controls were spotted on a second target. Targets were incubated for 3 or 4 h at 35 ± 1°C using a plastic transport box (Bruker Daltonik) serving as a humidity chamber in order to prevent the microdroplets from evaporating. After incubation, the remaining liquid was removed from the spots using filter paper, (size 37 × 100 mm, GE Healthcare GmbH, Freiburg im Breisgau, Germany). After overlaying the spots with 1 μl of α-cyano-4-hydroxycinnamic acid matrix including internal standard (MBT MASTeR prototype kit, Bruker Daltonik), MALDI-TOF MS spectra were acquired on a microflex smart instrument (Bruker Daltonik). The method was performed in triplicate.

#### Interpretation of Panel Results

##### Minimum inhibitory concentration

The spectra obtained from the MALDI-TOF MS acquisitions were processed using the MALDI Biotyper Software 3.1 (Bruker Daltonik), resulting in an identification score for each spot analyzed. A score ≥2.0 was considered as an indicator of bacterial growth detection. For each antimicrobial substance or substance combination, the minimum antibiotic concentration showing no bacterial growth, equivalent to a score <2.0, was defined as the MIC. In case all spots of one dilution series displayed scores <2.0, the MIC was defined as ≤0.25. MICs were calculated in triplicate, as the assay was simultaneously performed on three identical targets for each strain. The median MIC (middle value in ascending order) was used for further analysis.

##### Screening and confirmation

The MICs of cephalosporins determined in absence and presence of ESBL and/or AmpC inhibitor were compared in order to detect synergy, which was considered to be present when a ≥eightfold reduction of the MICs was observed after addition of clavulanic acid and/or cloxacillin. The results obtained for each cephalosporin were interpreted using a computer-based algorithm following the EUCAST criteria (EUCAST, 2017), obtaining three possible results: ESBL, AmpC, ESBL+AmpC or negative (Figure 1B).

### Detection of Resistance Genes

A genotypic characterization of all tested strains was performed with the PCR microarray Check-MDR CT103 XL (Check-Points, Wageningen, Netherlands). For an overview of the genes detected see Supplementary Table S4.

### Additional Phenotypic Diagnostic Methods

#### Broth Microdilution

The screening panel was reproduced on microtiter plates by performing BMD according to the guidelines of CLSI (2018) and the International Organization for Standardization (ISO) (ISO, 2006). In short, bacterial suspension and antibiotic solutions employed in the DOT-MGA were pipetted onto a first plate (30 μl of each solution, total volume of 60 μl), using a second one for the growth and sterile controls. Both microtiter plates were incubated for 18 ± 2 h at 35 ± 1°C. The MIC was interpreted as the lowest antibiotic concentration at which complete growth inhibition was seen. All MIC determinations were performed in triplicate. Median values were calculated for further analysis.

#### Combination Disk Test (CDT)

The following combination disk tests (Mast Diagnostica GmbH, Reinfeld, Germany) were carried out following the procedure recommended by the manufacturer: D63C (cefpodoxime alone and combined with clavulanic acid), D67C (cefpodoxime, cefotaxime and ceftazidime alone and combined with clavulanic acid) and D69C (cefpodoxime alone and combined with AmpC inducer, clavulanic acid and cloxacillin). Briefly, bacterial suspensions with a density equivalent to 0.5 McFarland standard was spread on Mueller-Hinton agar plates (BD GmbH, Heidelberg, Germany). Disks were then placed onto the inoculated medium, leaving enough space for inhibition zones to be seen correctly. Plates were then incubated at 35–37°C for 18 h, after which the diameter of the zones of inhibition was measured and recorded according to the instructions of use. The results of the three tests were interpreted as shown in Supplementary Table S1.

### Statistical Analysis

According to EUCAST’s guidelines (EUCAST, 2017), genotypic testing is the conclusive method to identify resistance mechanisms in cases where phenotypic techniques do not provide clear results. Thus, we considered the PCR an imperfect reference standard (Valenstein, 1990) in order to determine the positive and negative percent agreements (PPA and NPA, respectively) of the DOT-MGA, BMD, and CDT, which were calculated according to the statistical guidance of the Food and Drug Administration (FDA, 2007).

## Results

In preliminary experiments, DOT-MGA was performed on four well-characterized reference strains recommended by EUCAST for detection of ESBL and AmpC production (EUCAST, 2017). After 3 h of incubation, the resistance mechanisms of two of three resistant microorganisms were correctly identified. A correct identification of all three resistant strains was possible after 4 h of incubation. At both time points, the assay yielded a negative result for a susceptible reference strain (Table 1).

**Table 1:** Detection performance of the DOT-MGA screening panel on reference strains recommended by EUCAST.

Strain	Known resistance mechanism	DOT-MGA screening panel result	
		Incubation time	
		3 h	4 h
*K. pneumoniae*	ESBL	ESBL	ESBL
ATCC 700603
*E. coli* CCUG 58543	AmpC	Negative	AmpC
*E. coli* CCUG 62975	ESBL + AmpC	ESBL + AmpC	ESBL + AmpC
*E. coli* ATCC 25922	None	Negative	Negative

A total of 50 clinical *Enterobacterales* isolates displaying resistance against third generation cephalosporins were tested in order to further evaluate the detection performance of DOT-MGA. The additional genotypic testing by PCR allowed the identification of an OXA-48-producing *K. pneumoniae* strain (Supplementary Table S3). For each strain, DOT-MGA was performed in two set-ups, with incubation times of three and 4 h, respectively. The results obtained were further analyzed and compared with those of the PCR (Supplementary Table S2). Here, it could be confirmed that 4 h of incubation are required for a reliable detection, as also indicated by the preliminary experiments on reference strains (Supplementary Table S2).

The different resistance mechanisms were detected by DOT-MGA (4-h incubation), BMD, CDT and PCR in the following number of isolates, respectively; ESBL, 17,19,18,18; AmpC, 19,22,15,18; ESBL+AmpC, 1,1,0,1; none, 13,8,17,13. A detailed overview of the results yielded by each method can be found in Supplementary Table S3.

The following positive and negative percent agreement values (PPA/NPA) were obtained for the detection of each resistance mechanism by DOT-MGA: ESBL, 94.44/100%; AmpC, 94.44/93.75% and ESBL+AmpC, 100/100%. Percent agreement values of BMD and CDT were also calculated and are presented in Table 2, which shows a comparative overview of the detection performance of all three methods.

**Table 2:** Detection performance of DOT-MGA, BMD, and CDT on clinical isolates compared to PCR.

Resistance mechanism	Detection method (incubation time)					
	DOT-MGA (4 h)		BMD (18 h)		CDT (4 h)	
	PPA	NPA	PPA	NPA	PPA	NPA
ESBL	94.4%	100%	100%	96.9%	94.4%	96.9%
AmpC	94.4%	93.8%	100%	87.8%	61.1%	87.5%
ESBL + AmpC	100%	100%	100%	100%	0.0%	100%

## Discussion

The proposed approach offers an “all-in-one” screening method following the recommendations of EUCAST. It allows testing for different resistance mechanisms in a single step and displays higher PPA and NPA values than CDT, a well-established phenotypic test commonly used in routine laboratories. Furthermore, it yielded results comparable to those of BMD, while requiring an incubation period 14 h shorter. Since this method is not based on the detection of hydrolytic β-lactam products, it bypasses the challenges faced by other MALDI-TOF MS-based approaches that rely on this principle.

DOT-MGA was able to identify the production of ESBL in 17 of 18 isolates identified as positive by PCR. The remaining isolate displayed an indeterminate DOT-MGA result. This seems to be related to factors inherent to the strain in question, given that no significant growth was detected after 4 h of incubation when the assay was performed. Hence, it was not possible to identify any synergistic effects and, thus, any resistance mechanisms. The production of ESBL by this strain was confirmed by BMD after 18 h of incubation. The results of CDT were inconclusive as all three kits were positive. False negative results for slow growing strains shows to be one limitation of our method, since it relies on bacterial growth as do several other rapid phenotypic approaches.

AmpC-producing isolates pose a major diagnostic challenge, as ESBL inhibitors such as clavulanic acid have no effect on AmpC enzymes, thus interfering with the identification of ESBL production (Philippon et al., 2002). This leads to a wrong classification of such microorganisms as “non-ESBL-producing” and therefore as not multidrug resistant. In the case of strains displaying both resistance mechanisms, ESBL remains masked although AmpC can be successfully detected. For this reason, a method for detection of ESBL must necessarily also allow the proper identification of AmpC production. In order to tackle this problem, we designed a double identification strategy: (i) AmpC detection with cefepime, an AmpC-stable cephalosporin (Figure 1A, row F) (Thomson, 2001); (ii) additional detection of masked ESBL with either cefepime plus clavulanic acid (Figure 1A, row G) or cefotaxime plus clavulanic acid and cloxacillin as AmpC inhibitor (Figure 1A, row H). This confers our approach a higher accuracy of detection for strains producing solely AmpC as well as for those showing combined resistance mechanisms. The discrepancies observed between DOT-MGA and PCR correspond to strains showing no resistance genes, but a phenotypic resistance pattern compatible with AmpC production, confirmed by BMD, as is the case of one *E. aerogenes* isolate. Despite the high accuracy of the PCR, this was employed in our study as imperfect reference standard (Valenstein, 1990). Since such methods are based on the amplification of certain target genes regardless of their phenotypic expression, they might yield positive results in strains showing no phenotypic correlation due to the non-expression of the resistance gene(s), as well as false negative results in strains with phenotypic signs of AmpC production, most likely mediated by genes not targeted and thus not detected.

The test has been designed as a complementary diagnostic tool that can be integrated to the routine laboratory workflow at different points, i.e., simultaneous to the standard susceptibility testing of *Enterobacterales* isolates on a regular basis, or as a confirmation once a resistance against third generation cephalosporines has been determined.

The proposed assay represents a promising alternative to the methods of detection of resistance mechanisms currently available. It yields reliable results in a short time, providing concrete evidence that could directly impact the decision-making process in the healthcare setting. This would have several implications such as a direct improvement of the clinical outcome, a more rational use of antibiotics and shorter reaction times in the context of hospital infection control.

DOT-MGA can potentially be adapted for commercial production. Possible strategies for the automation of the method include: (i) developing targets coated with lyophilized antibiotic substances according to the panel’s layout, requiring only the addition of bacterial suspension to the spots; (ii) programming a new function within the existing Biotyper software in order to analyze the growth scores of each spot, programming it to follow an algorithm based on fix criteria (Figure 1B) in order to yield a final diagnosis.

## Author Contributions

CC-M, EI, and KB designed the experiments. CC-M and EI performed the experiments. CC-M, EI, KS, and MK designed and analyzed specific MALDI-TOF MS instrument settings for experiments. CC-M, EI, KS, and KB analyzed the data. CC-M and EI wrote the manuscript with input from KS, MK, and KB. All authors reviewed and edited the manuscript.

## Conflict of Interest Statement

EI and KB are inventors of a pending patent, which is owned by the University of Münster and licensed to Bruker. KS and MK are employees of Bruker Daltonik GmbH. The remaining author declares that the research was conducted in the absence of any commercial or financial relationships that could be construed as a potential conflict of interest.
