# Supplementary material for: Rapid Detection of Extended-Spectrum β-Lactamases (ESBL) and AmpC β-Lactamases in Enterobacterales: Development of a Screening Panel Using the MALDI-TOF MS-Based Direct-on-Target Microdroplet Growth Assay
Source: Front Microbiol. 2019 Jan 24;10:13. doi: 10.3389/fmicb.2019.00013 (PMC6353820; doi:10.3389/fmicb.2019.00013)
Supplement: Supplementary file 1 [file Data_Sheet_1.PDF]

Rapid detection of extended-spectrum β-lactamases (ESBL) and AmpC β-lactamases in *Enterobacterales*: Development of a screening panel using the MALDI-TOF MS-based direct-on-target microdroplet growth assay

Carlos L. Correa-Martínez et al.

Table S1. Interpretation of commercial combination disk tests.

Resistance mechanism	D63C*	D67C*	D69C*
ESBL	+	+	+
ESBL + AmpC	+	-	+
AmpC	-	-	+
Negative	-	-	-

*D63C, cefpodoxime alone and combined with clavulanic acid; D67C, cefpodoxime, cefotaxime and ceftazidime alone and combined with clavulanic acid; D69C, cefpodoxime alone and combined with AmpC inducer, clavulanic acid and cloxacillin (according to the manufacturer's recommendation).

Inconsistent result combinations were classified as "Indeterminate".

Table S2. Detection performance of DOT-MGA on 50 clinical *Enterobacterales* isolates compared to PCR after 3 and 4 hours of incubation.

Resistance mechanism	3 hours		4 hours	
	PPA	NPA	PPA	NPA
ESBL	66.7%	96.9%	94.4%	100%
AmpC	33.3%	93.8%	94.4%	93.8%
ESBL + AmpC	0.0%	100%	100%	100%

Table S3. Resistance mechanisms detected by BMD, CDT, DOT-MGA (3 and 4 hours of incubation) and PCR in 50 clinical *Enterobacterales* isolates.

Isolate	BMD	CDT	DOT-MGA		PCR	
			3 h	4 h	Result	Detected gene(s)
H. alvei	AmpC	-	-	AmpC	AmpC	ACC
C. freundii	AmpC	-	-	-	-	-
K. pneumoniae	ESBL	ESBL	-	ESBL	ESBL	CTX-M15
K. oxytoca	-	I	-	-	-	-
K. pneumoniae	ESBL	ESBL	-	ESBL	ESBL	CTX-M15
K. ornithinolytica	-	-	-	-	-	-
S. marcescens	-	AmpC	-	-	-	-
E. cloacae complex	AmpC	AmpC	-	AmpC	AmpC	ACT/MIR
E. coli	ESBL	ESBL	ESBL	ESBL	ESBL	CTX-M15
E. cloacae complex	AmpC	AmpC	-	AmpC	AmpC	ACT/MIR
E. cloacae complex	AmpC	AmpC	-	AmpC	AmpC	ACT/MIR
E. cloacae complex	AmpC	AmpC	-	AmpC	AmpC	ACT/MIR
E. coli	ESBL	ESBL	-	ESBL	ESBL	CTX-M9
K. pneumoniae	ESBL	ESBL	ESBL	ESBL	ESBL	CTX-M15
E. coli	ESBL	I	-	I	ESBL	CTX-M15
K. oxytoca	-	I	-	-	-	-
E. coli	ESBL	ESBL	ESBL	ESBL	ESBL	CTX-M9
E. cloacae complex	AmpC	AmpC	-	AmpC	AmpC	ACT/MIR
E. coli	ESBL	ESBL	ESBL	ESBL	ESBL	CTX-M15
E. coli	ESBL	ESBL	ESBL	ESBL	ESBL	CTX-M15
E. coli	ESBL	ESBL	ESBL	ESBL	ESBL	CTX-M15
E. coli	ESBL	ESBL	-	-	-	-
E. coli	ESBL	ESBL	-	ESBL	ESBL	SHV 238S+240K
E. coli	ESBL	ESBL	ESBL	ESBL	ESBL	CTX-M9
E. cloacae complex	AmpC	AmpC	AmpC	AmpC	AmpC	ACT/MIR
E. cloacae complex	AmpC	AmpC	AmpC	AmpC	AmpC	ACT/MIR
K. pneumoniae	-	I	-	-	-	OXA-48

E. coli	ESBL	ESBL	ESBL	ESBL	ESBL	CTX-M9
E. coli	ESBL/ AmpC	I	I	ESBL/ AmpC	ESBL/ AmpC	CMY II/CTX-M15
E. aerogenes	AmpC	I	AmpC	AmpC	-	-
E. coli	ESBL	ESBL	ESBL	ESBL	ESBL	CTX-M15
E. coli	ESBL	ESBL	ESBL	ESBL	ESBL	CTX-M15
M. morganii	AmpC	AmpC	AmpC	AmpC	AmpC	DHA
E. coli	ESBL	ESBL	ESBL	ESBL	ESBL	CTX-M15
K. pneumoniae	-	-	-	-	-	-
C. koseri	ESBL	ESBL	ESBL	ESBL	ESBL	SHV 238S+240K
K. pneumoniae	ESBL	ESBL	-	ESBL	ESBL	CTX-M15
K. oxytoca	-	-	ESBL	-	-	-
C. freundii	AmpC	AmpC	-	AmpC	AmpC	CMY II
M. morganii	AmpC	I	AmpC	AmpC	AmpC	DHA
E. cloacae complex	AmpC	I	AmpC	AmpC	AmpC	ACT/MIR
M. morganii	AmpC	AmpC	-	AmpC	AmpC	DHA
E. aerogenes	AmpC	AmpC	AmpC	AmpC	-	-
E. cloacae complex	AmpC	AmpC	-	AmpC	AmpC	ACT/MIR
E. cloacae complex	AmpC	AmpC	-	-	-	-
E. cloacae complex	AmpC	I	-	AmpC	AmpC	ACT/MIR
C. freundii	AmpC	I	AmpC	AmpC	AmpC	CMY II
E. cloacae complex	-	AmpC	-	-	-	-
E. cloacae complex	AmpC	I	-	AmpC	AmpC	ACT/MIR
H. alvei	AmpC	I	-	-	AmpC	ACC

- : negative result.

I: indeterminate result (see also Table S1).

Table S4. β-lactamase genes detected by the PCR microarray.

Carbapenemases		ESBL				Minor ESBL	AmpC
GES*	OXA-24	CTX-M-1 group	CTX-M-9 group	TEM wt	SHV wt	BEL	ACC
GIM	OXA-48	CTX-M-1 subgroup	CTX-M-15 subgroup	TEM 104K	SHV 238A	GES**	ACT/MI R
IMP	OXA-58	CTX-M-2 group	CTX-M-25 group	TEM 164C	SHV 238S	PER	CMY I/MOX
KPC	VIM	CTX-M-3 subgroup	CTX-M-32 subgroup	TEM 164H	SHV 240K	VEB	CMY II
NDM	SPM	CTX-M-8 group		TEM 164S			DHA
OXA-23				TEM 238S			FOX

*GES enzymes with carbapenemase activity: 2, 4-6, 13-15, 17, 18, 20, 21.
**GES enzymes with ESBL activity: 1, 3, 7-12, 16, 19, 22.
